# Synthesis and Structural Evaluation of Organo-Ruthenium Complexes with β-Diketonates

**DOI:** 10.3390/molecules22020326

**Published:** 2017-02-20

**Authors:** Matija Uršič, Tanja Lipec, Anton Meden, Iztok Turel

**Affiliations:** Faculty of Chemistry and Chemical Technology, University of Ljubljana, Večna pot 113, 1001 Ljubljana, Slovenia; matija.ursic@fkkt.uni-lj.si (M.U.); tanja90@gmail.com (T.L.); Anton.Meden@fkkt.uni-lj.si (A.M.)

**Keywords:** organoruthenium complexes, β-diketonate ligands, halogen substituents, 1,3,5-triazaphosphoadamantane (pta) ligand, disorder

## Abstract

Four novel ruthenium organometallic complexes: [(η^6^-*p*-cymene)Ru(4,4,4-trifluoro-1-(4-bromophenyl)-1,3-butanedione)Cl] (**1**), [(η^6^-*p*-cymene)Ru(4,4,4-trifluoro-1-(4-bromophenyl)-1,3-butanedione)pta]PF_6_ (**2**), [(η^6^-*p*-cymene)Ru(4,4,4-trifluoro-1-(4-iodophenyl)-1,3-butanedione)Cl] (**3**) and [(η^6^-*p*-cymene)Ru(4,4,4-trifluoro-1-(4-iodophenyl)-1,3-butanedione)pta]PF_6_ (**4**) were synthesized and characterized by elemental analysis, infrared (IR), UV-Vis, NMR and mass spectroscopy and single-crystal X-ray diffraction. The crystal structures and spectroscopic data were compared to the previously published complexes [(η^6^-*p*-cymene)Ru(4,4,4-trifluoro-1-(4-chloro-phenyl)-1,3-butanedione)Cl] (**5**) and [(η^6^-*p*-cymene)Ru(4,4,4-trifluoro-1-(4-chlorophenyl)-1,3-butanedione)pta]PF_6_ (**6**). The pairs of complexes **1** and **3** as well as **2** and **4** are isostructural, with the former crystallizing in triclinic P-1 and the latter in monoclinic P2_1_/c. The ruthenium(II) ion is found in a pseudo-octahedral “piano-stool” geometry in all compounds. Bond lengths and angles are consistent with other complexes of this type. Complexes **2** and **4** exhibit some moderate dynamic disorder. The lack of hydrogen bonding and major π-π interactions means that most of intramolecular interactions are fairly weak and involve halogen atoms present. This was further confirmed by ^1^H-NMR spectra, where a significant difference is observed only on the ligand near the halogen atom, following an expected trend. The combined data show that the difference in any activity depends substantially on the type of the ligand′s substituted halogen atom.

## 1. Introduction

**Figure 1:**
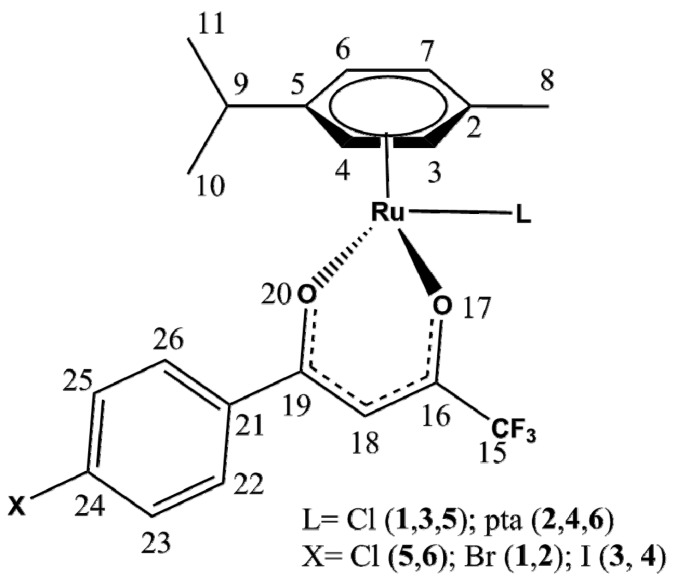
General structure of the studied complexes with the crystallographic atom numbering scheme, used throughout this text.

Metal complexes exert many interesting properties which find diverse applications in numerous aspects of human life, among which their biological effects are the most intensely studied [1]. One of the reasons that the biological activities of metal-based compounds have been a rather hot topic in recent times is their potential use as anticancer agents. Within this group, ruthenium-based complexes are among the most promising, with several lead compounds such as the NAMI-A [2], KP1019 [3] and RAPTA complexes [4]. Unlike biological activity, catalytical properties of ruthenium complexes have been firmly established and extensively used in organic chemistry, especially in olefin metathesis [5,6,7,8,9]. Our group has previously studied catalytically and biologically active complexes with various diketonates. They have exhibited their potential for use as catalysts for ortho arylation via C–H activation [10], as well as anticancer activity on CH1 ovarian cancer line in the low micromolar range. The biological activity was significantly increased by the substitution of chloride with the monodentate phosphine ligand 1,3,5-triazaphosphoadamantane (pta), which also increases water solubility and substantially slows down hydrolysis [4]. The complexes [(η^6^-*p*-cymene)Ru(4,4,4-trifluoro-1-(4-chlorophenyl)-1,3-butanedione)Cl] (**5**) and [(η^6^-*p*-cymene)Ru(4,4,4-trifluoro-1-(4-chlorophenyl)-1,3-butanedione)pta]PF_6_ (**6**) were the ones that showed the highest activity against the CH1 ovarian cancer line among similar compounds tested. The same two compounds also showed moderate activity in MG63 osteosarcoma cancer line tests, while **5** was previously tested as a catalyst for ortho arylation and showed a moderate conversion rate and high selectivity [4,10,11]. These two complexes were identified as good lead compounds due to their high activity and good potential for fine-tuning. Commercial availability of various analogous ligands and the relative ease of preparing additional non-commercial derivatives was also considered. To start with the systematic evaluation of this class of compounds, two pairs of bromo and iodo analogues, **1**, **2** and **3**, **4** (Figure 1) were synthesized and characterized with the aim of identifying the impact of the relatively minor substitution of the ligand-bound halogen atom on the physico-chemical properties of the complex and possible emergent structural elements or trends that might help to predict and explain possible structure-activity relationship in future biological and catalytical testing.

## 2. Results

### 2.1. Synthesis and Spectroscopic Analysis

All metal complexes have been synthesized using previously established protocols [10,11] and purified with silica column chromatography. ^1^H-, ^19^F- and ^31^P-NMR and CHN analysis were used to determine the purity of the compounds. Additionally, HRMS was used to determine the composition, which was finally confirmed by single crystal X-ray diffraction. Single crystals used for structure determination were grown from solutions by slow evaporation of solvent from a mixture of acetone and heptane. All compounds were also characterized by IR and UV-Vis spectroscopy. Selected spectra of all the methods are attached as supplementary information.

The NMR spectra of all new compounds are in line with the previously known compounds and other analytical methods. The spectra of compounds **1** and **3** were recorded in CDCl_3_, while those of **2** and **4** had to be recorded in deuterated acetone, as they are barely soluble in deuterated chloroform. The ^1^H-NMR spectra of **1**–**4** display all the expected signals of the coordinated *p*-cymene, *β*-diketonate and, where applicable, pta. The ligand′s phenyl ring hydrogen atoms H22–H26 can be found in the region between 7.5–8 ppm in the form of two doublets followed by the signals of the *α*-protons (H18) around 6.13 for chloride complexes and 6.73 ppm for pta complexes. The cymene aromatic ligands are in the area between 5.3–5.6 for **1** and **3** and 6.3–6.4 for **2** and **4** as multiplets. The pta signals are two 3-proton doublets around 4.5 ppm and a six-proton singlet at 4.3 ppm. The characteristic cymene single-proton septet of H9 is found near 2.9 ppm for chlorido and 2.8 ppm for pta compounds, with the latter slightly overlapping the residual water peak (2.85 ppm) in deuterated acetone. The last two signals of H8 and H10-H11 can be found as a singlet near 2.10 ppm and a double doublet near 1.35 ppm, respectively. The ^31^P-NMR spectra for **2** and **4** contain one singlet belonging to pta in the region around −28.7 ppm, which is strongly shifted to higher frequencies with respect to the corresponding free ligand at –102.50 ppm. Such a deshielding effect is a common consequence of coordination to an electropositive metal ion. Both spectra also contain a typical septet around 144 ppm for the PF_6_^−^ counter ion. Moreover, in the ^19^F spectrum, we can observe a single peak belonging to the CF_3_ group on the ligand at 74.2 ppm for **1** and **3** and 74.9 ppm for **2** and **4**, while the latter two also feature a doublet belonging to PF_6_^−^ counterion at 72.5 ppm. The NMR spectra of all the new compounds are very similar to each other and to previously isolated compounds, which were used to assign the peaks without recording 2D spectra [10,11]. The only real differences are, as expected, minor (less than 0.1 ppm) shifts of the signals belonging to the hydrogens on the phenyl ring of the ligand between the comparable compounds, which is due to the effect of different halide substituents. The shift is significantly larger in complexes **5** and **6**, which agrees with the established theory of halogens as electron-withdrawing groups well (see Supplementary materials).

In the IR spectra, characteristic bands for ν(C=O) and ν(C=C) modes in bidentate O,O-donor diketonate ligands appear in the range from around 1575 to 1515 cm^−1^. Spectra of complexes **1**–**4** showed a slight shift in those peaks, as they were placed in the area between 1600 and 1463 cm^–1^, which is characteristic for coordination on a metal atom. Aromatic ν(C–H) bands were weak and mostly overlapping with diketone stretching bands in the range from 1600 and 1450 cm^−1^. Intense absorption bands in the frequency range 1350−1100 cm^−1^ correspond to C−F bonds, while the strong peak in the spectra of compounds **2** and **4** around 833 cm^−1^ was assigned to ν(P–F) in the counterion [12].

The high resolution ESI mass spectra of complexes **1** and **3** showed the presence of one major peak corresponding to [(η^6^-*p*-cymene)Ru(β-diketonate)]^+^ (M − Cl), while **2** and **4** showed the presence of two major peaks, the more intense one corresponding to the [(η^6^-*p*-cymene)Ru(β-diketonate)(pta)]^+^ species (M − PF_6_) and the less intense peak assignable to [(η^6^-*p*-cymene)Ru(β-diketonate)]^+^ (M − PF_6_ − pta).

UV-Vis spectra for all compounds were recorded from 250–800 nm in methanol and show a single, strong and fairly wide peak between 310 and 320 nm, with a rather minor, wide and hardly noticeable peak, which blends itself with the tail from the bigger 310 nm peak, at approximately 380 nm. By analyzing previously published TD-DFT calculations of complexes with similar ligands, we can infer that the major peak near 310 nm arises due to a series of metal-to-ligand charge transfer transitions, coupled with some interligand and ligand-centered transitions. The absorbance tail near 380 nm should arise mainly due to metal-to-ligand charge transfer transitions and some contributions from d-d transitions [13,14,15].

### 2.2. X-ray Crystal Structures

**Figure 2:**
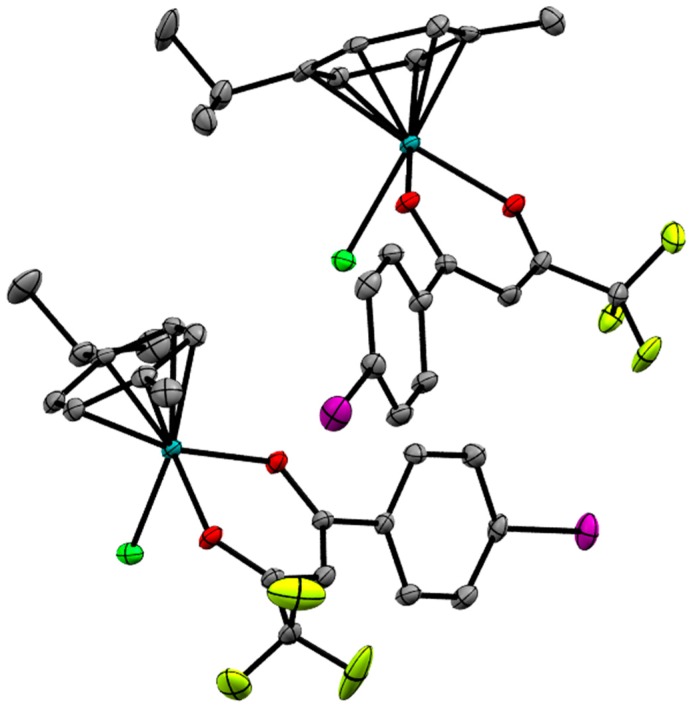
Asymmetric unit of **3**. Thermal ellipsoids are drawn at 30% probability. Hydrogen atoms are omitted for clarity. Compounds **1** and **3** are isostructural.

**Figure 3:**
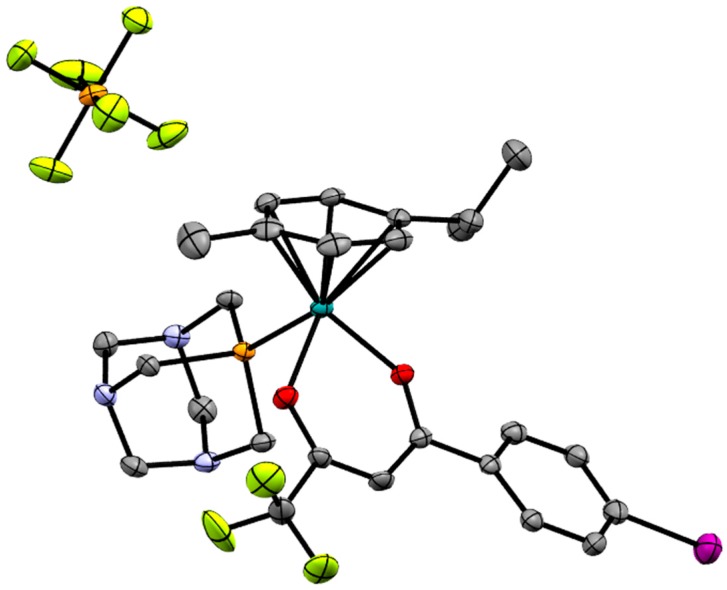
Asymmetric unit of **4**. Thermal ellipsoids are drawn at 30% probability. Hydrogen atoms and atoms introduced to model disorder are omitted for clarity. Compounds **2** and **4** are isostructural.

All four new compounds differ a fair bit from the already reported complexes **5** and **6**, with the pairs of compounds **1** and **3** as well as **2** and **4** being isostructural. The coordination geometry of all the complexes is best described as a pseudo-octahedral “piano-stool”, with the cymene ligand being the “stool top” and three coordination sites being occupied by a O,O-chelate and one monodentate ligand around the Ru(II) center. The neutral chlorido analogues **1** and **3** both crystallize in P-1 triclinic space group with two molecules in the asymmetric unit, while **5** is found in P2_1_/n with only one molecule in the asymmetric unit (Figure 2). The pta complexes share a bit more similarities. They all crystallize in the same Laue class, with **2** and **4** being found in P2_1_/n and **6** in C2/c, with one cationic molecule of the complex and one molecule of the hexafluoridophosphate counterion per asymmetric unit in all three of them (Figure 3). Bond lengths between ruthenium and the monodentate ligand are slightly shorter than average for similar complexes, with Ru–Cl bonds in **1**, **3** and **5** ranging from 2.392 Å to 2.405 Å and Ru–P bonds for **2** and **4** between 2.314 Å and 2.318 Å, with **6** being even shorter at 2.297 Å. The Ru–O bond length in all the complexes except **5** is slightly dependent on both the monodentate ligand and the orientation of the O,O-ligand. The bond nearer to the -CF_3_ group is a bit longer, especially in **1**, **3** and **6**, while the pta complexes **2** and **4** feature more equal bond lengths and the bond lengths in complex **5** are completely equal to each other (Table 1). The bidentate ligand creates a six-membered ring containing ruthenium ion in which the bonds are delocalized, with C–O bond lengths ranging between 1.252 Å to 1.283 Å and C–C bonds between 1.369 Å and 1.421 Å, with the shorter bond always lying closer to the -CF_3_ group and the difference between bonds about 0.03 Å. This delocalization is an important feature in functionality of β-diketonato compounds [16,17,18,19,20]. The O–Ru–O angles show minor strain as they are between 86.67° and 88.10°. All of the selected and remaining bond and angle values are consistent with other previously reported complexes of a similar type [10,11,21,22,23,24,25].

**Figure 4:**
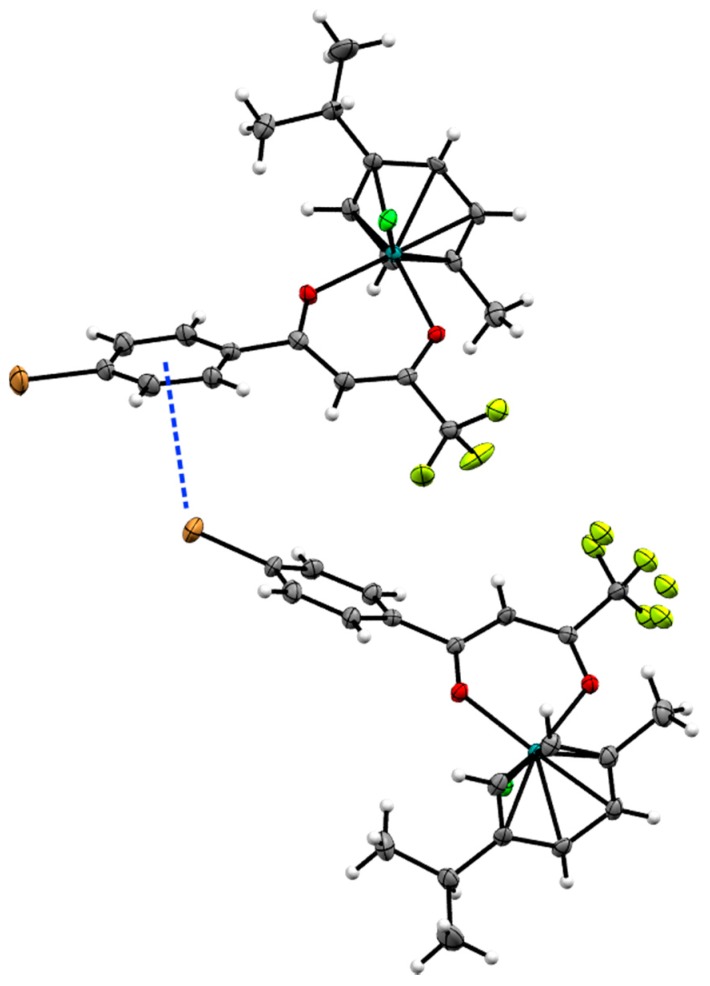
Interaction between the bromine atom and phenyl ring in **1**. Thermal ellipsoids drawn at 30% probability. Centroid to halogen atom distances: **1**: 3.772 Å; **3**: 3.807 Å.

**Figure 5:**
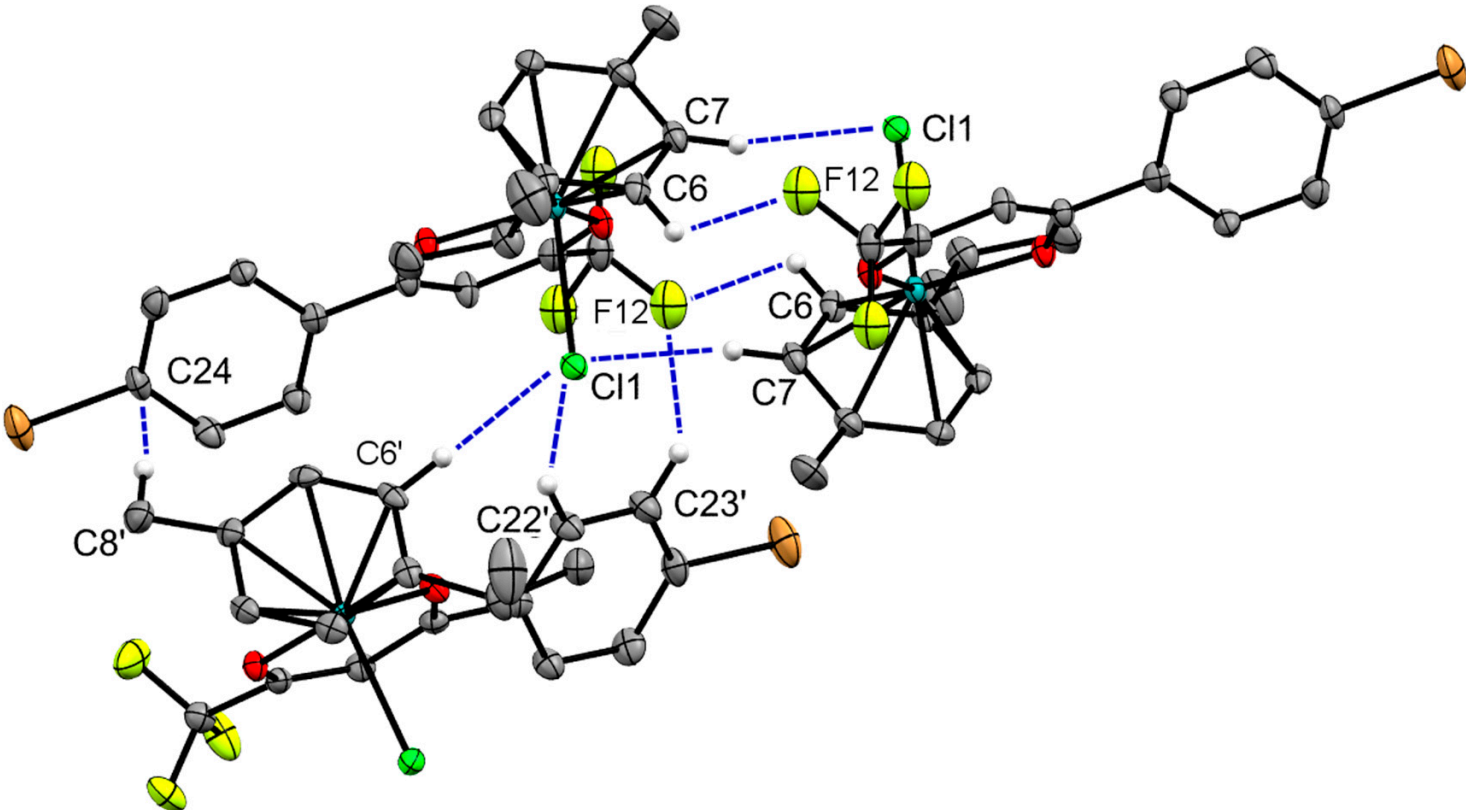
Weak interactions in **1** with the participating non-hydrogen atoms labelled. Thermal ellipsoids drawn at 30% probability. Hydrogen atoms not participating in marked interactions omitted for clarity.

The structures exhibit no classical hydrogen bonds; however, weak interactions between halogen atoms and aromatic hydrogens are present in all of the compounds, while we encounter additional interactions between hydrogen atoms and fluorine atoms of the hexafluoridophosphate counterions in the pta complexes **2**, **4** and **6**. A weak, but notable interaction (centroid to halogen atom distance: **1**: 3.772 Å; **3**: 3.807 Å) is observed between the halogen atom on the phenyl ring and the ligands’ phenyl ring of a crystallographically independent molecules in **1** and **3** (Figure 4), as this seems to be the cause of significantly different packing as compared to **5**, where no such interaction is present. Another interesting thing to note is that the cell volume difference of 16 Å^3^ between **1** and **3** is mainly due to a 13 Å^3^ solvent inaccessible void appearing in **3**. The supramolecular structures of **1** and **3** are characterized by layers running across the b-c diagonal formed by interactions of the halogen atoms with the aromatic hydrogen atoms of cymene and the O,O-ligand, while **5** has a 3D structure with nothing of much note. Each chloride in **1** and **3** interacts with a cymene aromatic hydrogen of a symmetrically equivalent molecule, forming a dimer about the center of inversion, which is also supported by interactions between the -CF_3_ group and other cymene and ligand aromatic hydrogens to form a 3D zig-zag motif (Figure 5).

**Figure 6:**
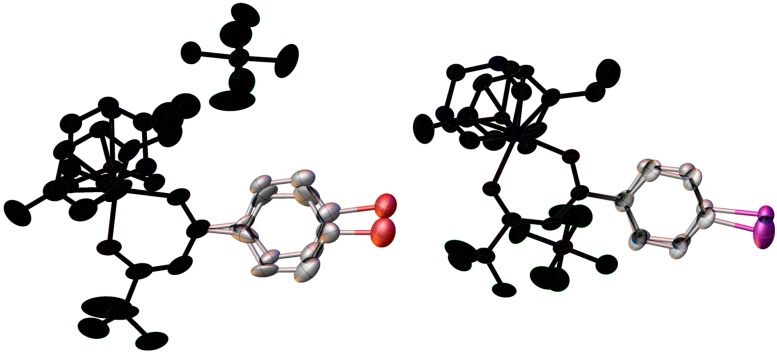
Disorder in **2** (**left**) and **4** (**right**). Darkness indicates overlap between two possible structures, black meaning complete overlap. Hydrogen atoms omitted for clarity. Thermal ellipsoids at 50% probability. Occupancy ratios: **2**—0.659/0.341, **4**—0.777/0.223.

Crystal structures of pta complexes **2** and **4** show moderate dynamic disorder of the phenyl ring of the ligand, which was satisfactorily modelled by splitting the ring over two positions (Figure 6). The model is suggesting dynamic “waving” of C19–C21 bond extending to the rest of the phenyl ring due to lack of major interactions between the atoms on the ring and the rest of the structure. This is supported by the difference in disorder magnitude between otherwise very similar structures. The bromine atom in **2** forms no interactions, as opposed to the iodine atom in **4** forming a weak interaction with hydrogen atoms on C10, leading to lower disorder occupancy (0.223 compared to 0.341 in **2**) and significantly less observed movement of the phenyl ring (C18–C19–C21 angle change of 2° in **4** compared to 11° in **2**).

The supramolecular structures of **2** and **4** are mainly stabilized by interactions between hydrogen and fluorine atoms of the hexafluoridophosphate anion (distances between 2.348 Å and 2.646 Å), with the molecules forming layers in the b-c plane. The protruding -CF_3_ groups don’t form close interactions with each other or any other atom. Compound **6** features additional interactions involving pta nitrogen and cymene aromatic hydrogen atoms (distances of 2.641 Å and 2.658 Å), as well as one involving the chloride atom and a counterion fluorine (3.121 Å), resulting in a different packing that creates voids with the volume of approximately 80 Å^3^, with inner solvent accessible surface of 9 Å^3^. Additional figures are available in the Supplementary materials.

## 3. Experimental Section

### 3.1. Materials and Methods

[(η^6^-*p*-Cymene)RuCl(μ-Cl)]_2_ was purchased from Strem Chemicals (Strem Chemicals Inc., Newburyport, MA, USA), the β-diketonates were purchased from Fluorochem (Derbyshire, UK) and the rest of the chemicals were obtained from Sigma-Aldrich (St. Louis, MO, USA). All the materials were used as received. ^1^H-NMR spectra were recorded on an Avance III 500 spectrometer (Bruker Corporation, Billerica, MA, USA) at room temperature and 500.10 MHz by using TMS as an internal standard. ^19^F- and ^31^P-NMR spectra were recorded on the same instrument at 470 and 202 MHz respectively. Infrared spectra were recorded with a Spectrum 100 FTIR spectrometer (PerkinElmer Inc., Waltham, MA, USA), equipped with a Specac Golden Gate Diamond ATR as a solid sample support. UV-vis spectra were collected on Perkin-Elmer LAMBDA 750 UV/vis/near-IR spectrophotometer. Elemental analyses were recorded using a Perkin-Elmer 2400 II instrument (CHN), and HRMS were measured on a 6224 Accurate Mass TOF LC/MS instrument (Agilent Technologies, Santa Clara, CA, USA).

### 3.2. Crystal Structure Determination

X-ray diffraction data for compounds **1**–**4** (Table 2) were collected on an Oxford Diffraction SuperNova diffractometer with Mo (**1**,**2**,**4**) or Cu (**3**) microfocus X-ray source (Kα radiation, λ_Mo_ = 0.71073 Å, λ_Cu_ = 1.54184 Å) with mirror optics and an Atlas detector at 150(2) K. The structures were solved in Olex^2^ graphical user interface [26] by direct methods implemented in SHELXT and refined by a full-matrix least-squares procedure based on F^2^ using SHELXL [27]. All non-hydrogen atoms were refined anisotropically. The hydrogen atoms were placed at calculated positions and treated using appropriate riding models. Dynamic disorder in **2** and **4** was modelled using two-positional disorder, using SHELX afix constraints for the phenyl ring due to the instability of the refinement without it. Additionally, SHELX eadp constraint was used for the disordered phenyl ring of compound **4**. The programs Mercury, Olex^2^, Mogul, ORTEP, and Platon were used for data analysis and figure preparation [21,26,28,29,30]. CCDC No. 1524059 for compound **1**, 1524062 for compound **2**, 1524060 for compound **3** and 1524061 for compound **4** contains the supplementary crystallographic data for this paper. These data can be obtained free of charge via http://www.ccdc.cam.ac.uk/conts/retrieving.html (or from the CCDC, 12 Union Road, Cambridge CB2 1EZ, UK; Fax: +44 1223 336033; E-mail: deposit@ccdc.cam.ac.uk).

### 3.3. Synthesis

**Scheme 1:**
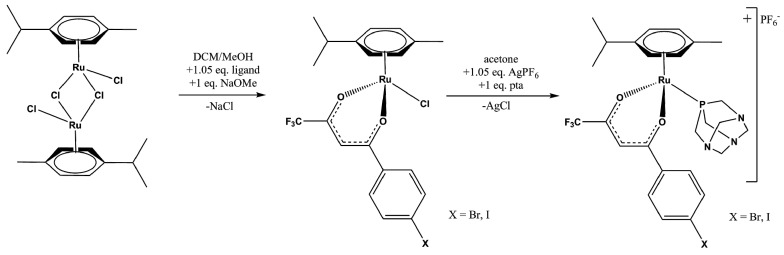
General scheme for two-step preparation of complexes.

The complexes were prepared by the method illustrated in Scheme 1.

#### 3.3.1. Synthesis of **1** and **3**

Compounds **1** and **3** have been synthesized by the previously published procedure [10]. [(η^6^-*p*-Cymene)RuCl(μ-Cl)]_2_ (40.0 mg, 0.065 mmol), the appropriate β-diketonate (2.4 equiv.), and sodium methoxide, as a base (2.2 equiv.), were dissolved in a 10/1 dichloromethane/methanol mixture (20 mL). The ligands 4,4,4-trifluoro-1-(4-bromophenyl)-1,3-butanedione and 4,4,4-trifluoro-1-(4-iodo-phenyl)-1,3-butanedione were used in these reaction procedures. The reaction mixture was refluxed for 4 h. The solvent was then removed, and dichloromethane was added to precipitate the byproduct NaCl and other insoluble impurities, which were subsequently filtered through Celite powder. The solution was concentrated to 1–2 mL, and approximately 10 mL of *n*-hexane was added, until the product started to precipitate. The solution was then left to stand for 20 min and the product was filtered and left to dry at 45 °C for 4 h. Purification on a chromatographic column with silica gel as the stationary phase and a mixture of dichloromethane and methanol (7% methanol in dichloromethane) was performed, if proven necessary by ^1^H-NMR analysis. *R*_f_ factors of all compounds are between 0.85 and 0.90, while [(η^6^-*p*-cymene)RuCl(μ-Cl)]_2_ as a common impurity has an *R*_f_ factor of 0.2.

*[(η^6^-p-Cymene)Ru(4,4,4-trifluoro-1-(4-bromophenyl)-1,3-butanedione)Cl]* (**1**). Yield: 71%. ^1^H-NMR (500 MHz, CDCl_3_): δ 7.72 (d, *J* = 8.6 Hz, 2H, H23,H25), 7.55 (d, *J* = 8.6 Hz, 2H, H22, H26), 6.13 (s, 1H, H18), 5.61 (dd, *J* = 14.6, 5.8 Hz, 2H, Ar-H cym), 5.36–5.32 (m, 2H, Ar-H cym), 2.94 (sep, *J* = 6.9 Hz, 1H, Ar-C*H*(CH_3_)_2_ cym), 2.29 (s, 3H, Ar-CH_3_ cym), 1.38 (dd, *J* = 6.9, 4.1 Hz, 6H, Ar-CH(C*H*_3_)_2_ cym). ^19^F-NMR (471 MHz, CDCl_3_): δ −74.22 ppm (C15F_3_). Selected IR bands (cm^−1^, ATR): 3052, 2965, 1600, 1584, 1561, 1309, 1296, 1196, 1182, 1140, 1008, 784, 664. ESI-HRMS (CH_3_CN) *m*/*z*: found (calcd.) 530.9551 (530.9543) [M(^81^Br) − Cl]^+^, 528.9553 (528.9564) [M(^79^Br) − Cl]^+^. Elemental analysis CHN (%) for C_20_H_19_BrClF_3_O_2_Ru: theoretical C, 42.53; H, 3.39; experimental C, 42.33; H, 3.40. UV-vis (λ [nm] (ε [L·mol^−1^·cm^−1^]) *c* = 0.5 × 10^−4^ mol/L, MeOH): 310 (24030).

*[(η^6^-p-Cymene)Ru(4,4,4-trifluoro-1-(4-iodophenyl)-1,3-butanedione)Cl]* (**3**). Yield: 67%. ^1^H-NMR (500 MHz, CDCl_3_): δ 7.76 (d, *J* = 8.5 Hz, 2H, H23,H25), 7.56 (d, *J* = 8.5 Hz, 2H, H22,H26), 6.12 (s, 1H, H18), 5.61 (dd, *J* = 14.9, 5.8 Hz, 2H, Ar-H cym), 5.36–5.32 (m, 2H, Ar-H cym), 2.94 (sep, *J* = 7.0 Hz, 1H, Ar-C*H*(CH_3_)_2_ cym), 2.29 (s, 3H, Ar-CH_3_ cym), 1.38 (dd, *J* = 6.9, 3.8 Hz, 6H, Ar-CH(C*H*_3_)_2_ cym). ^19^F-NMR (471 MHz, CDCl_3_): δ −4.22 ppm (C15F_3_). Selected IR bands (cm^−1^, ATR): 3050, 2965, 1598, 1581, 1555, 1529, 1310, 1297, 1250, 1195, 1183, 1141, 1072, 1004, 784, 728, 659. ESI-HRMS (CH_3_CN) *m*/*z*: found (calcd.) 576.9422 (576.9425) [M − Cl]^+^. Elemental analysis CHN (%) for C_20_H_19_ClF_3_IO_2_Ru: theoretical C, 39.26; H, 3.13; experimental C, 39.54; H, 3.20. UV-vis (λ [nm] (ε [L·mol^−1^·cm^−1^]) *c* = 0.5 × 10^−4^ mol/L, MeOH): 320 (30660).

#### 3.3.2. Synthesis of **2** and **4**

Compounds **2** and **4** have been synthesized by the previously published procedure [11]: The appropriate chlorido complex **1** or **3** (0.10 mmol) was mixed with pta (0.11 mmol, 1.1 equiv.) and AgPF_6_ (0.11 mmol, 1.1 equiv.) in acetone and left to stir 48 h without heating. The precipitated salt was filtered through Celite powder. The solution was concentrated and addition of *n*-heptane (approx. 25 mL) caused the precipitation of the product as fine yellow powder upon standing for 15–30 min. The products were filtered and left to dry at 45 °C for 4 h. Purification on a chromatographic column with silica gel as the stationary phase and a mixture of DCM and methanol (10% methanol in DCM) was performed, if proven necessary by ^1^H-NMR analysis. *R*_f_ factors of compounds **2** and **4** are between 0.35 and 0.50, while the corresponding starting complexes (**1** and **3**) as a common impurity have *R*_f_ factor between 0.85 and 0.95.

*[(η^6^-p-Cymene)Ru(4,4,4-trifluoro-1-(4-bromophenyl)-1,3-butanedione)pta]PF_6_* (**2**). Yield: 91%. ^1^H-NMR (500 MHz, Acetone-*d*_6_) δ 8.05 (d, *J* = 8.7 Hz, 2H, H23, H25), 7.79 (d, *J* = 8.6 Hz, 1H, H22, H26), 6.74 (s, 1H, H18), 6.42 (d, *J* = 6.2 Hz, 1H, Ar-H cym), 6.37–6.27 (m, 3H, Ar-H cym), 4.68–4.61 (m, 3H, NC*H*_2_N pta), 4.58 (d, *J* = 13.4 Hz, 3H, NC*H*_2_N pta), 4.44 (q, *J* = 2.2 Hz, 6H, PC*H*_2_N pta), 2.77 (sep, *J* = 6.9 Hz, 1H, Ar–C*H*(CH_3_)_2_ cym), 2.13 (s, 3H, Ar-C*H*_3_ cym), 1.36 (dd, *J* = 19.5, 6.9 Hz, 6H, Ar-CH(C*H*_3_)_2_ cym). ^19^F-NMR (471 MHz, Acetone-*d*_6_) δ −72.49 (d, *J* = 707.6 Hz, PF_6_). −74.94(C15F_3_). ^31^P-NMR (202 MHz, Acetone) δ −28.69 (P-pta), between −134.17 and −154.41 (m) (PF_6_). Selected IR bands (cm^−1^, ATR): 2925, 1599, 1583, 1559, 1537, 1483, 1445, 1421, 1393, 1311, 1296, 1243, 1200, 1149, 1113, 1099, 1076, 1041, 1009, 973, 947, 899, 876, 833, 786, 736, 670, 648, 627. ESI-HRMS (CH_3_CN) *m*/*z*: found (calcd.) 688.0316 (688.0312) [M(^81^Br) − PF_6_]^+^, 686.0330 (686.0333) [M(^79^Br) − PF_6_]^+^, 530.9550 (530.9554) [M (^81^Br) − PF_6_ − pta]^+^, 528.9560 (528.9564) [M (^79^Br) − PF_6_ − pta]^+^. Elemental analysis CHN (%) for C_26_H_31_BrF_9_N_3_O_2_P_2_Ru: theoretical C, 37.56; H, 3.76; N, 5.05; experimental C, 37.38; H, 3.69; N, 4.87. UV-vis (λ [nm] (ε [L·mol^−1^·cm^−1^]) *c* = 1 × 10^−3^ mol/L, MeOH): 310 (15250).

*[(η^6^-p-Cymene)Ru(4,4,4-trifluoro-1-(4-iodophenyl)-1,3-butanedione)pta]PF_6_* (**4**). Yield: 87%. ^1^H-NMR (500 MHz, Acetone-*d*_6_) δ 7.99 (d, *J* = 8.5 Hz, 2H, H23, H25), 7.87 (d, *J* = 8.5 Hz, 2H, H22, H26), 6.72 (s, 1H, H18), 6.41 (d, *J* = 6.2 Hz, 1H, Ar-H cym), 6.32 (dt, *J* = 14.5, 6.5 Hz, 3H, Ar-H cym), 4.63 (d, *J* = 13.1 Hz, 3H NC*H*_2_N pta), 4.58 (d, *J* = 13.4 Hz, 3H, NC*H*_2_N pta), 4.44 (s, 6H PC*H*_2_N pta), 2.76 (sep, *J* = 6.9 Hz, 1H, Ar-C*H*(CH_3_)_2_ cym), 2.12 (s, 3H, Ar-C*H*_3_ cym), 1.35 (dd, *J* = 19.2, 6.9 Hz, 6H, Ar-CH(C*H*_3_)_2_ cym). ^19^F-NMR (471 MHz, Acetone-*d*_6_) δ −72.54 (d, *J* = 707.5 Hz, PF_6_). −74.94(C15F_3_). ^31^P-NMR (202 MHz, Acetone) δ −28.74 (P-pta), between −127.82 and −158.15 (m) (PF_6_). Selected IR bands (cm^−1^, ATR): 2924, 1597, 1579, 1554, 1535, 1480, 1443, 1390, 1312, 1296, 1242, 1196, 1151, 1112, 1099, 1075, 1056, 1040, 1005, 973, 946, 899, 876, 833, 784, 739, 666, 625. ESI-HRMS (CH_3_CN) *m*/*z*: found (calcd.) 734.0187 (734.0194) [M]^+^, 576.9420 (576.9420) [M − pta]^+^. Elemental analysis CHN (%) for C_26_H_31_F_9_IN_3_O_2_P_2_Ru: theoretical C, 35.55; H, 3.56; N, 4.78; experimental C, 35.75; H, 3.48; N, 4.66. UV-vis (λ [nm] (ε [L·mol^−1^·cm^−1^]) *c* = 1 × 10^−3^ mol/L, MeOH): 320 (18860).

## 4. Conclusions

Using established synthesis protocols, four novel organometallic ruthenium diketonate complexes were synthesized, fully characterized and their crystal structures determined. The structures were compared with two already known ruthenium diketonates and several differences in the corresponding crystal structures and NMR spectra were observed. The most notable difference is the interaction between the bromide and iodide atoms with the phenyl rings on the ligands that even if weak, considerably changes the packing of complexes **1** and **3** and could also be important for biological or catalytic properties. Other things to note are the fairly strong influence of the -CF_3_ group, visible from the Ru–O, C–O and C–C bond lengths near it, and the interactions with the counterion in cationic complexes. The latter could prove to be the most important in further research, as it has been found previously that the same compounds with different counterions have slightly different ^1^H-NMR peak shifts, which shows that some intramolecular interactions between the counterion and the complex are present, even in solution [23]. Our NMR data shows the electron-withdrawing effect of halido substituents on the ligand’s phenyl ring, fitting the shifts expected from established theory well—the two signals indicating the aromatic hydrogen atoms are the most split in the *para*-chlorido substituted complexes, followed by bromide and iodide. The combined data show that the difference in any activity depends substantially on the type of the ligand´s substituted halogen atom.

## Figures and Tables

**Table 1:** Selected bond lengths (Å) and angles (°) in the studied compounds **1**–**6**. All bond lengths and angles for compounds **1**–**4** can be found in supplementary information in respective tables.

Bond/Angle	1	3	5	2	4	6
Ru1–O17	2.096(3)	2.097(4)	2.091(2)	2.081(8)	2.081(7)	2.094(4)
Ru1’–O17’	2.095(3)	2.099(4)	/	/	/	
Ru1–O20	2.081(3)	2.079(3)	2.092(1)	2.074(8)	2.074(6)	2.072(4)
Ru1’–O20’	2.076(2)	2.088(4)	/	/	/	/
Ru1–Cl1	2.393(1)	2.396(1)	2.405(1)	/	/	/
Ru1’–Cl1’	2.394(1)	2.397(1)	/	/	/	/
Ru1–P30	/	/	/	2.318(2)	2.314(2)	2.3270(7)
O17–Ru1–O20	87.7(1)	88.1(2)	87.44(3)	87.6(3)	87.6(2)	87.44(7)
O17’–Ru1’–O20’	87.3(1)	88.0(2)	/	/	/	/

**Table 2:** Crystal data of complexes **1**–**6**.

Complex Number	1	2	3	4
Empirical formula	C_20_H_19_BrClF_3_O_2_Ru	C_26_H_31_BrF_9_N_3_O_2_P_2_Ru	C_20_H_19_ClF_3_IO_2_Ru	C_26_H_31_F_9_IN_3_O_2_P_2_Ru
Formula weight	564.78	831.46	611.77	878.45
Temperature/K	150.00(10)	150.00(10)	150.00(10)	150.00(10)
Crystal system	triclinic	monoclinic	triclinic	monoclinic
Space group	P-1	P2_1_/c	P-1	P2_1_/c
a/Å	10.0249(4)	10.6584(5)	9.9973(3)	10.6889(4)
b/Å	12.9431(6)	14.8139(6)	12.9283(5)	14.8208(5)
c/Å	18.0374(7)	19.6695(8)	18.2806(8)	19.8820(6)
α/°	72.014(4)	90	71.413(4)	90
β/°	80.020(3)	94.661(4)	80.368(3)	93.538(3)
γ/°	76.696(4)	90	76.830(3)	90
Volume/Å^3^	2153.05(17)	3095.4(2)	2169.09(14)	3143.65(17)
Z	4	4	4	4
ρ_calc_·g/cm^3^	1.742	1.784	1.873	1.856
μ/mm^−1^	2.746	1.986	18.506	1.666
F (000)	1112.0	1656.0	1184.0	1728.0
Radiation	Mo Kα (λ = 0.7107)	Mo Kα (λ = 0.7107)	Cu Kα (λ = 1.5418)	Mo Kα (λ = 0.7107)
Reflections collected	16,610	17,899	15,274	20,267
Independent reflections	9837 (R_int_ = 0.0328, R_sigma_ = 0.0668)	8116 (R_int_ = 0.0400, R_sigma_ = 0.0660)	8508 (R_int_ = 0.0529, R_sigma_ = 0.0637)	8291 (R_int_ = 0.0345, R_sigma_ = 0.0529)
Data/restraints/parameters	9837/0/509	8116/0/440	8508/0/511	8291/0/410
Goodness-of-fit on F^2^	1.007	1.031	1.040	1.030
Final R indexes (I ≥ 2σ (I))	R_1_ = 0.0445, wR_2_ = 0.0909	R_1_ = 0.0665, wR_2_ = 0.1699	R_1_ = 0.0445, wR_2_ = 0.1217	R_1_ = 0.0532, wR_2_ = 0.1059
Final R indexes (all data)	R_1_ = 0.0753, wR_2_ = 0.1027	R_1_ = 0.1132, wR_2_ = 0.1942	R_1_ = 0.0548, wR_2_ = 0.1325	R_1_ = 0.0869, wR_2_ = 0.1207
Largest diff. peak/hole/e Å^−3^	0.94/−1.03	1.64/−0.89	1.59/−1.29	1.55/−0.73

## Supplementary Materials

Supplementary materials are available online.
