# Supplementary material for: Synthesis and Structural Evaluation of Organo-Ruthenium Complexes with β-Diketonates
Source: Molecules. 2017 Feb 20;22(2):326. doi: 10.3390/molecules22020326 (PMC6155601; doi:10.3390/molecules22020326)
Supplement: Supplementary file 1 [file molecules-22-00326-s001.pdf]

Supplementary information

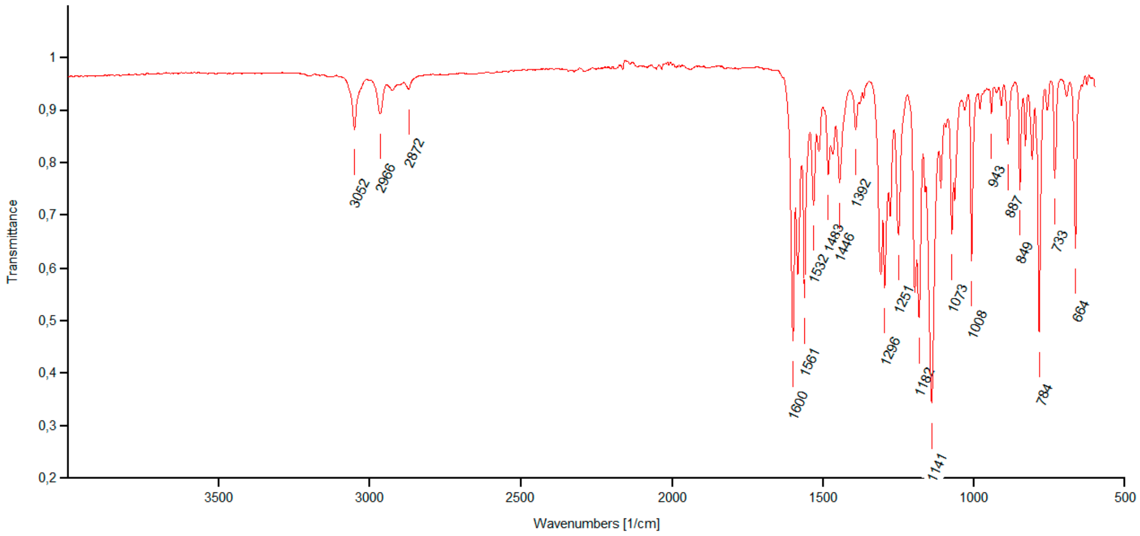

Figure 1. IR spectrum of [(η^6-*p*-cymene)Ru(4,4,4-trifluoro-1-(4-bromophenyl)-1,3-butanedione)Cl], **1**.

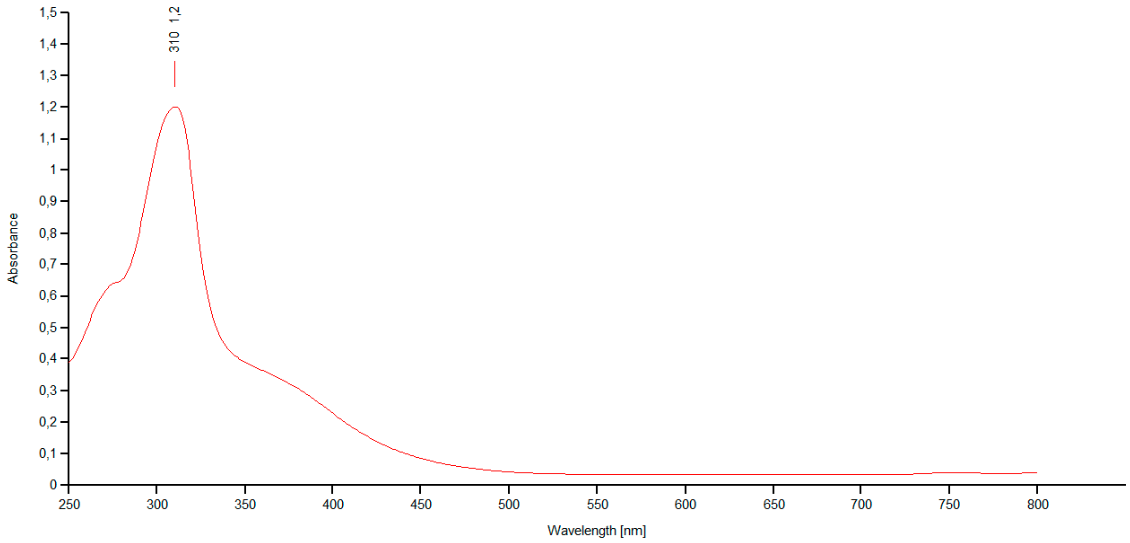

Figure 2. UV-Vis spectrum of [(η^6-*p*-cymene)Ru(4,4,4-trifluoro-1-(4-bromophenyl)-1,3-butanedione)Cl], **1.**

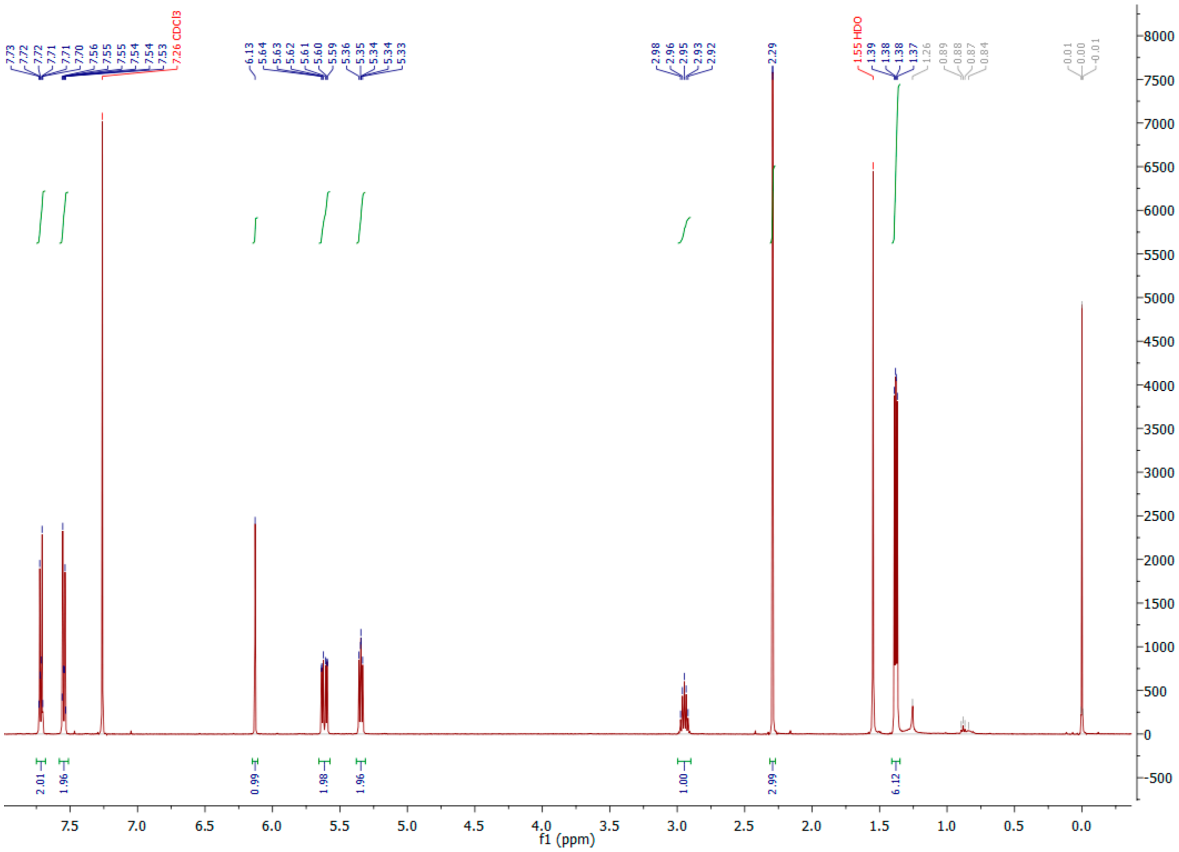

Figure 3. NMR spectrum of [(η[6]-*p*-cymene)Ru(4,4,4-trifluoro-1-(4-bromophenyl)-1,3-butanedione)Cl], **1**.

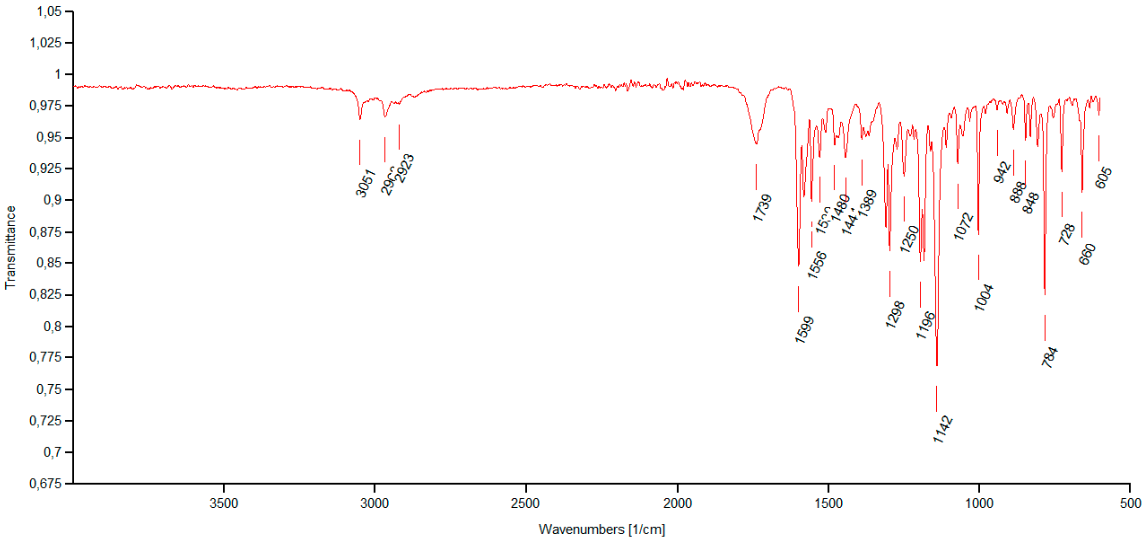

Figure 4. IR spectrum of [(η[6]-*p*-cymene)Ru(4,4,4-trifluoro-1-(4-iodophenyl)-1,3-butanedione)Cl], **3**.

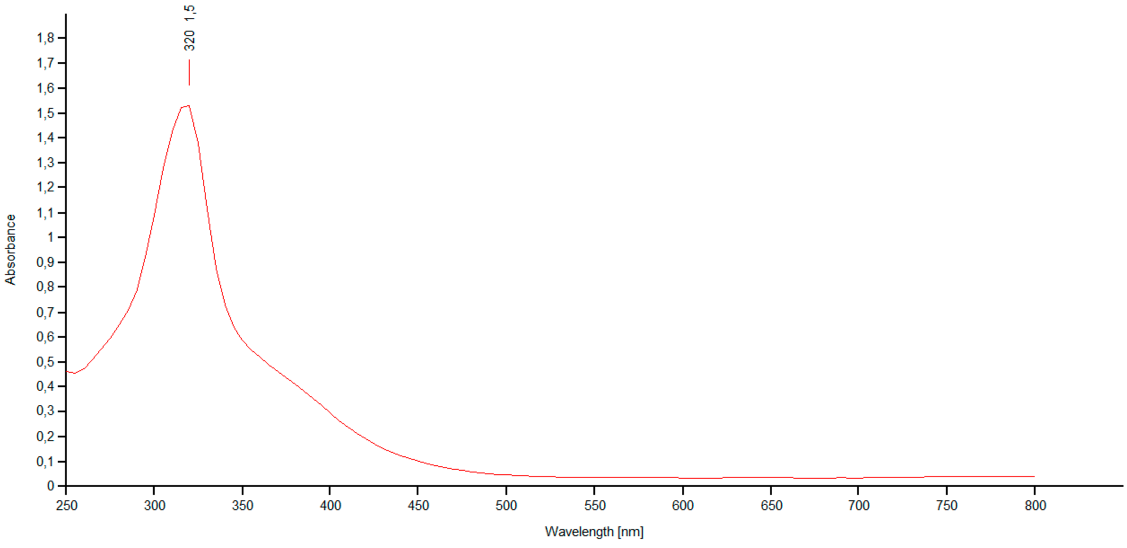

Figure 5. UV-Vis spectrum of [(η^6-*p*-cymene)Ru(4,4,4-trifluoro-1-(4-iodophenyl)-1,3-butanedione)Cl], **3**.

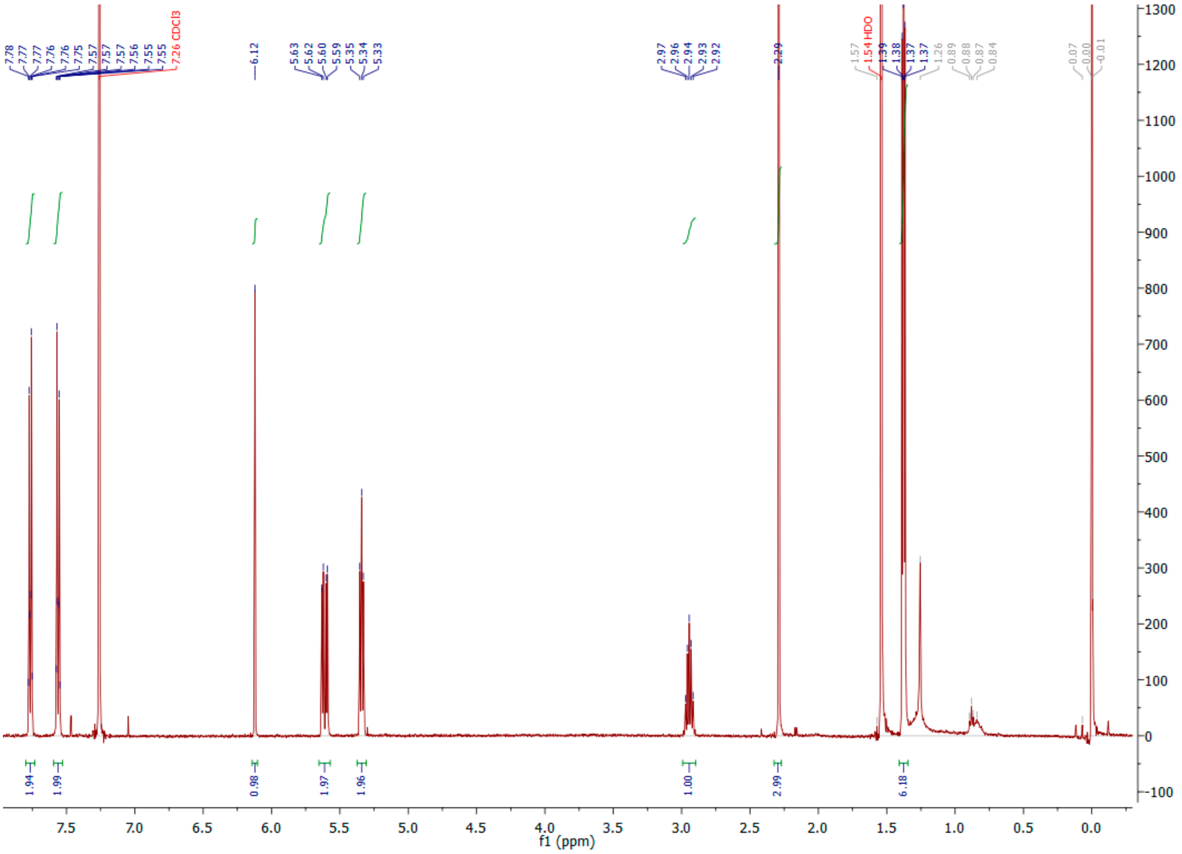

Figure 6. NMR spectrum of [(η^6-*p*-cymene)Ru(4,4,4-trifluoro-1-(4-iodophenyl)-1,3-butanedione)Cl], **3**.

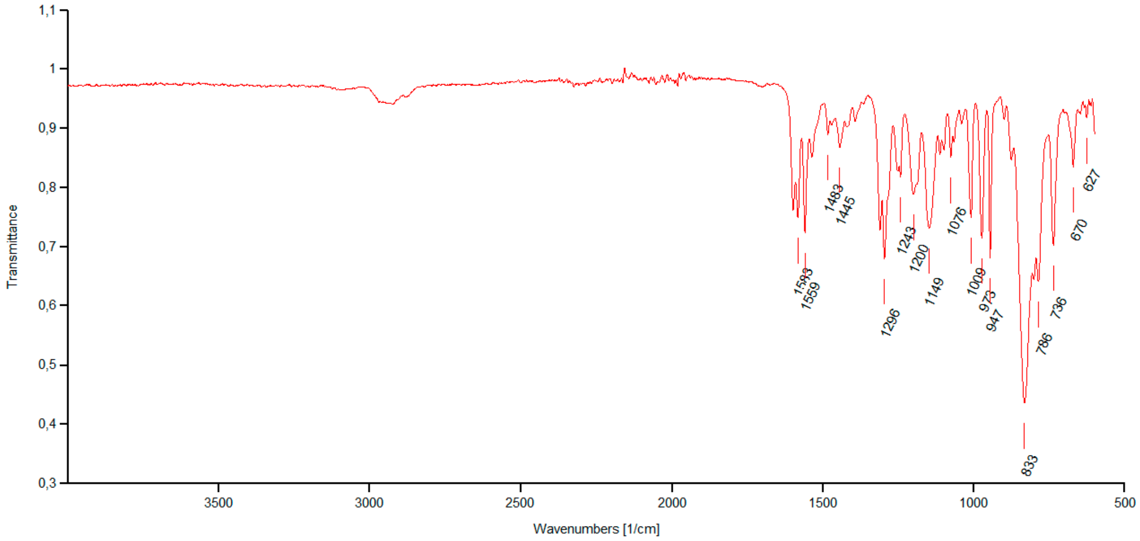

Figure 7. IR spectrum of [(η^6-*p*-cymene)Ru(4,4,4-trifluoro-1-(4-bromophenyl)-1,3-butanedione)pta]PF_6, **2**.

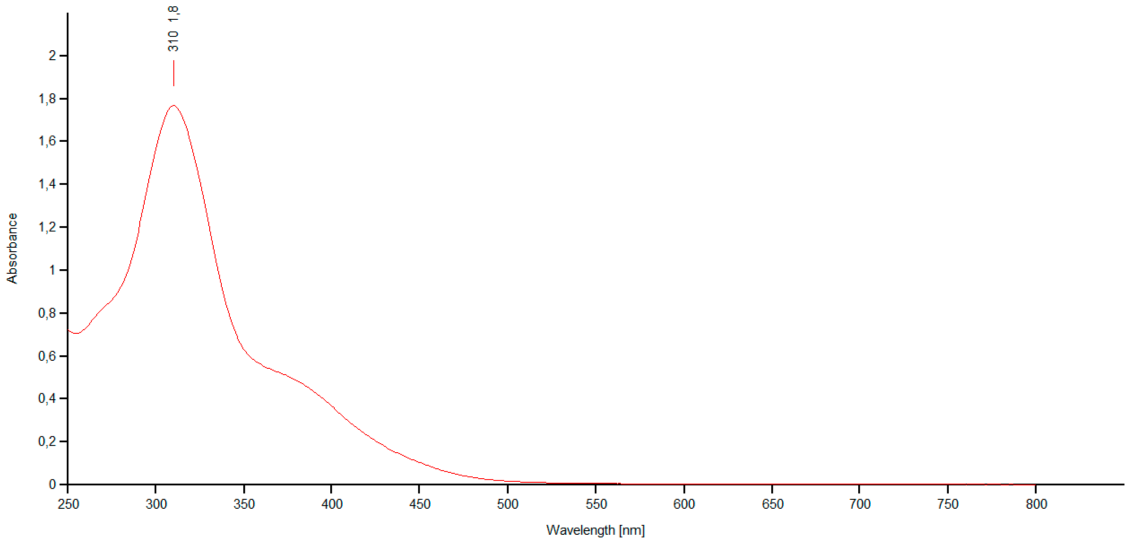

Figure 8. UV-Vis spectrum of [(η^6-*p*-cymene)Ru(4,4,4-trifluoro-1-(4-bromophenyl)-1,3-butanedione)pta]PF_6, **2**.

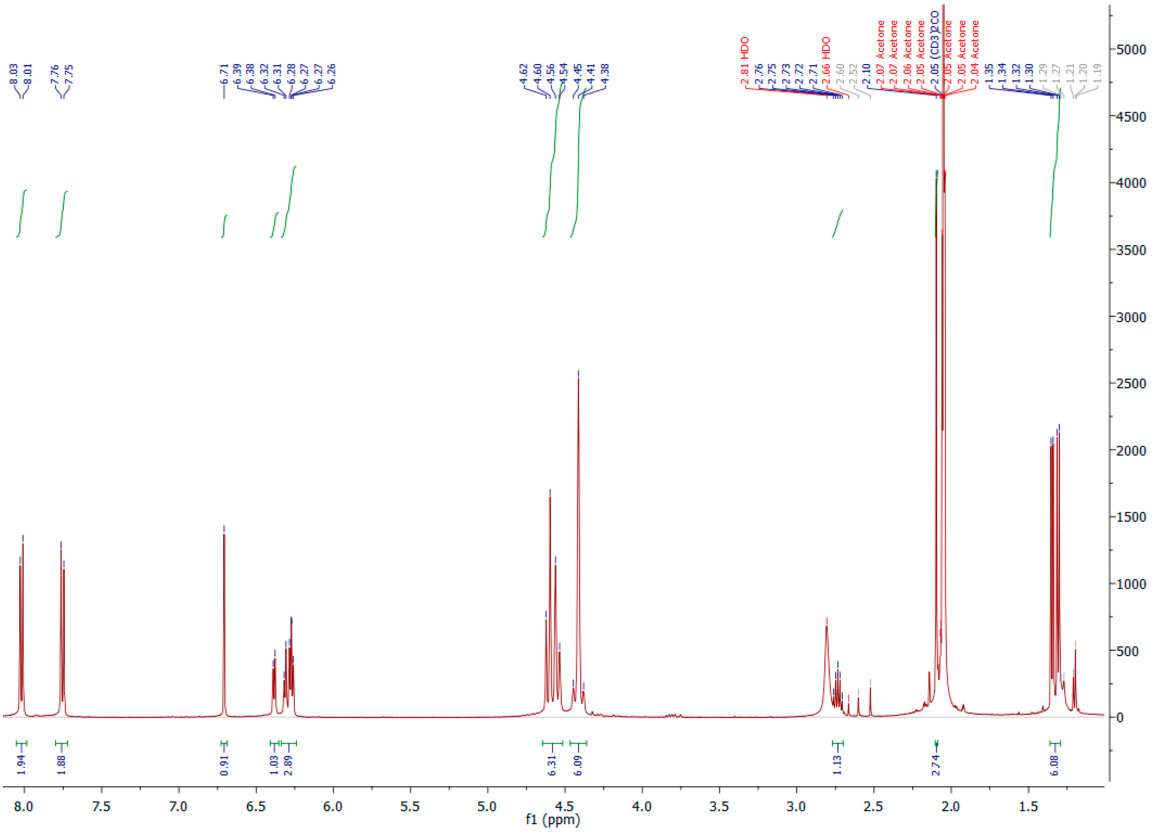

Figure 9. NMR spectrum of [(η⁶-*p*-cymene)Ru(4,4,4-trifluoro-1-(4-bromophenyl)-1,3-butanedione)pta]PF_6, **2**.

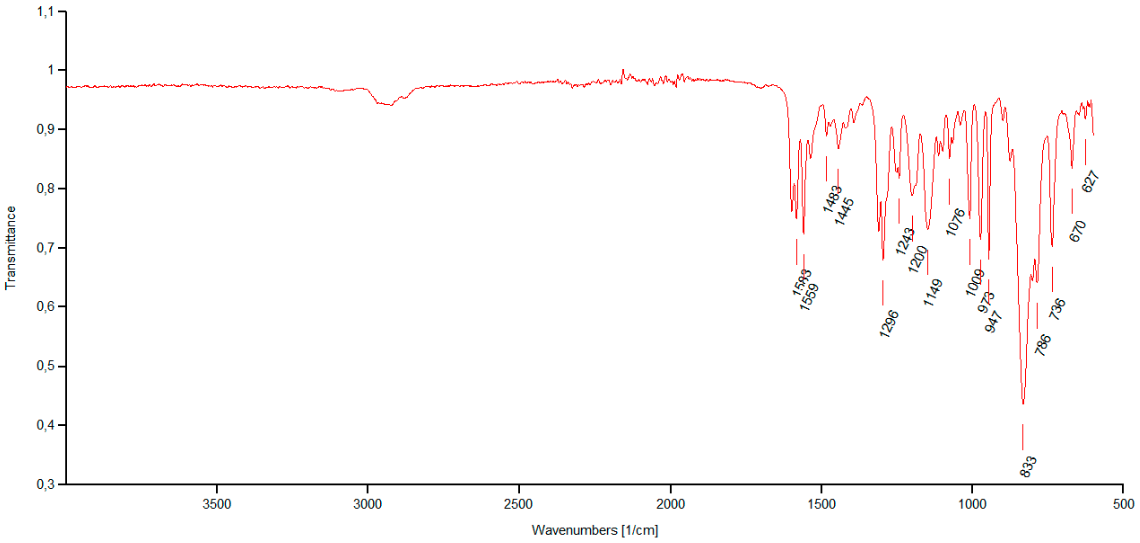

Figure 10. IR spectrum of [(η⁶-*p*-cymene)Ru(4,4,4-trifluoro-1-(4-iodophenyl)-1,3-butanedione)pta]PF_6, **4**.

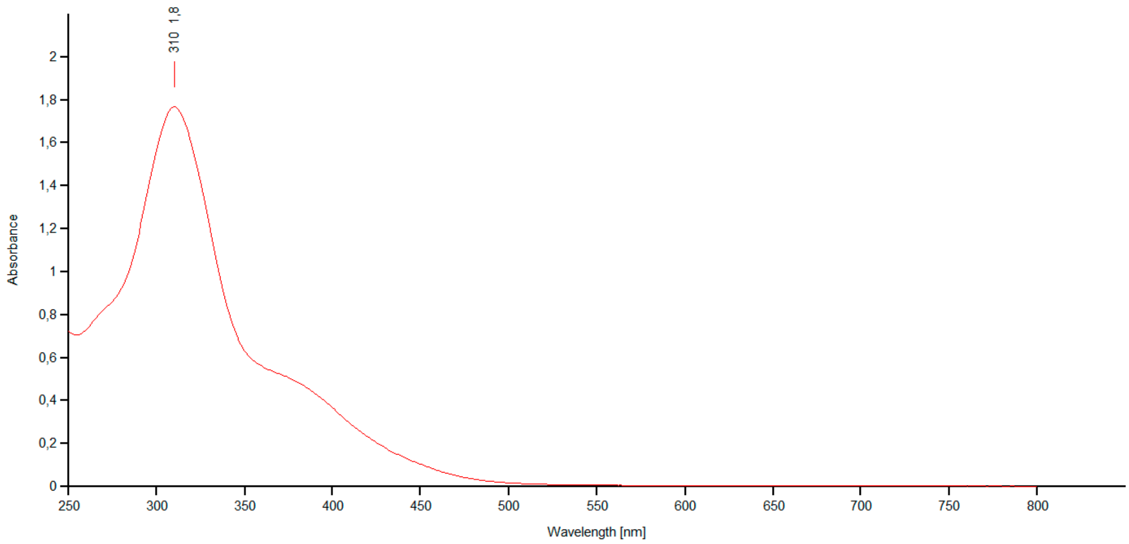

Figure 11. UV-Vis spectrum of [(η[6]-*p*-cymene)Ru(4,4,4-trifluoro-1-(4-iodophenyl)-1,3-butanedione)pta]PF_6, **4.**

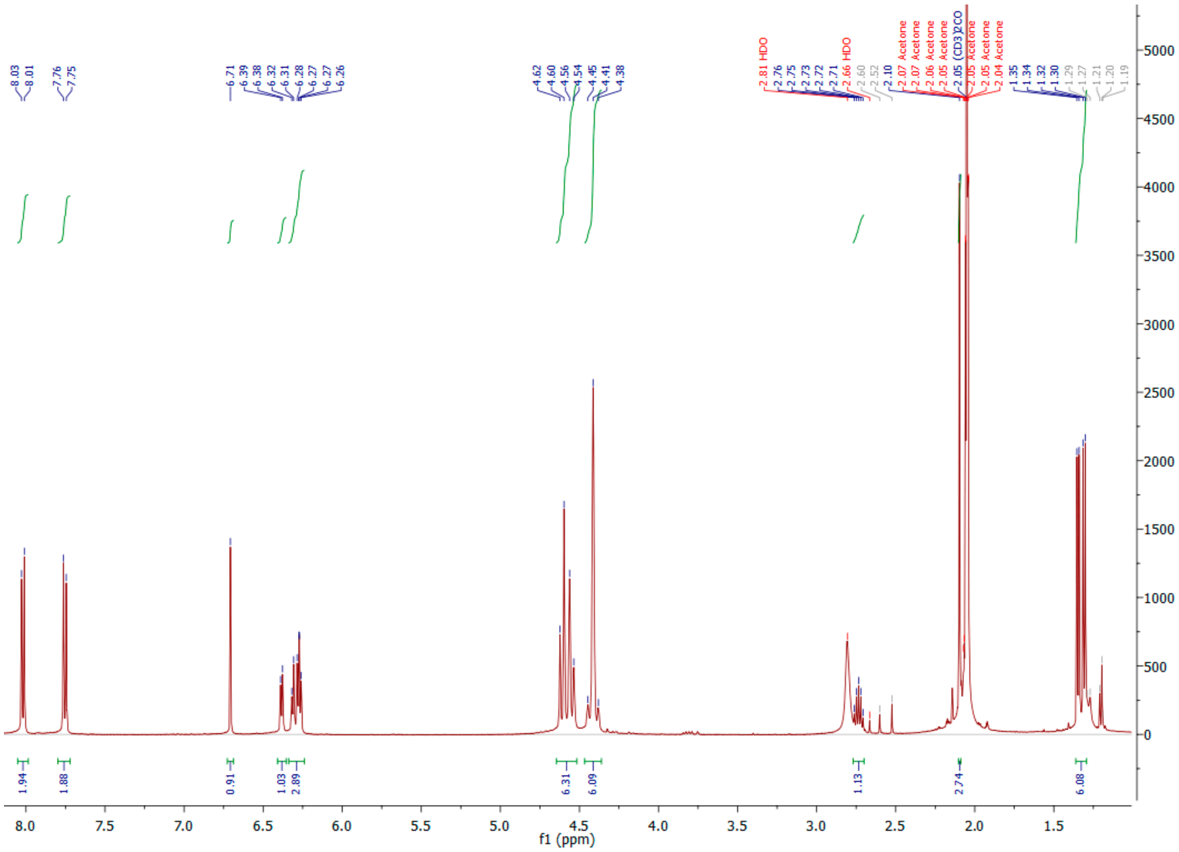

Figure 12. IR spectrum of [(η[6]-*p*-cymene)Ru(4,4,4-trifluoro-1-(4-iodophenyl)-1,3-butanedione)pta]PF_6, **4**.

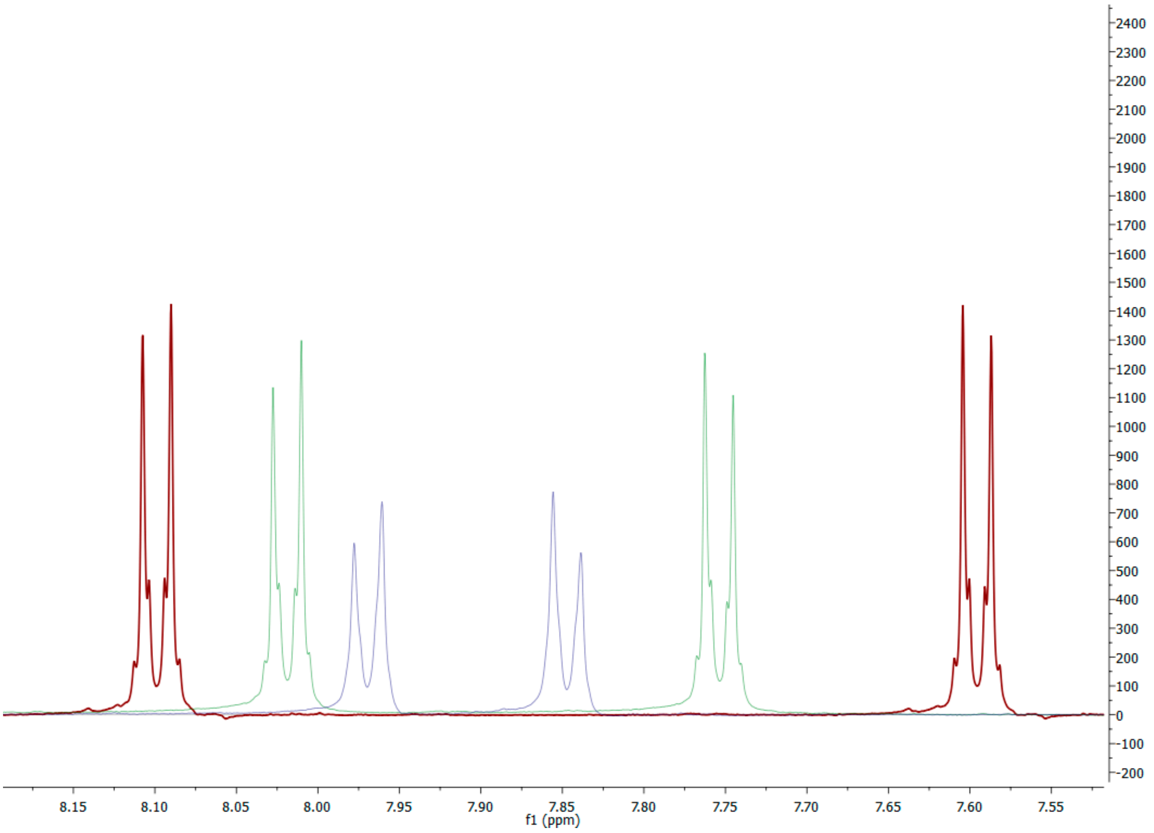

Figure 13. ^{1}H-NMR shifts of the hydrogen atoms on the phenyl ring of the ligand for complexes **2** (green), **4** (blue) and **6** (red).

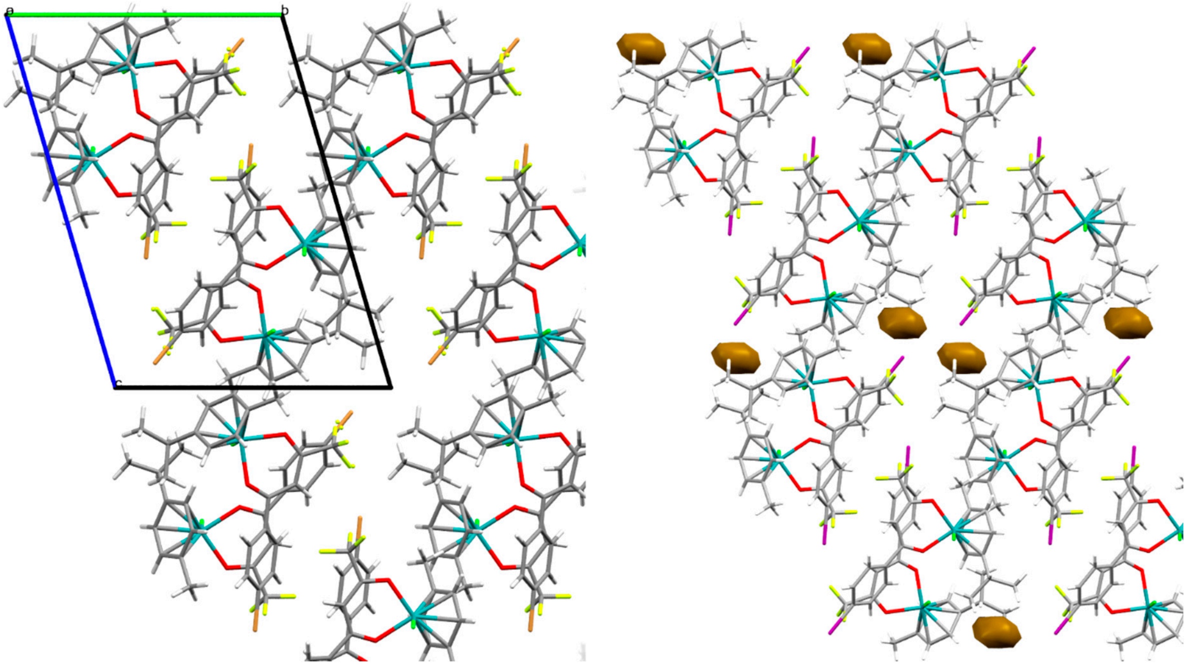

Figure 14. Packing of **1** (left) and **3** (right). Voids are indicated by ochre clouds.

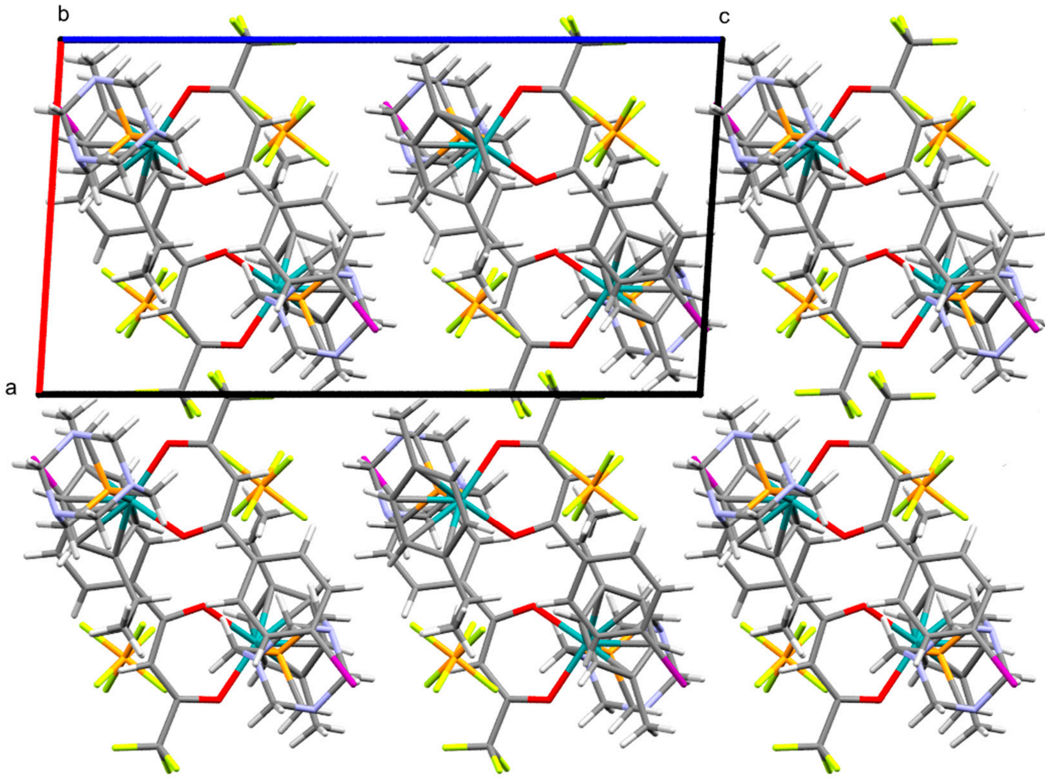

Figure 15. Packing of **4**. Note that the protruding -CF_3 groups are not in the same plane and do not interact with any atoms.

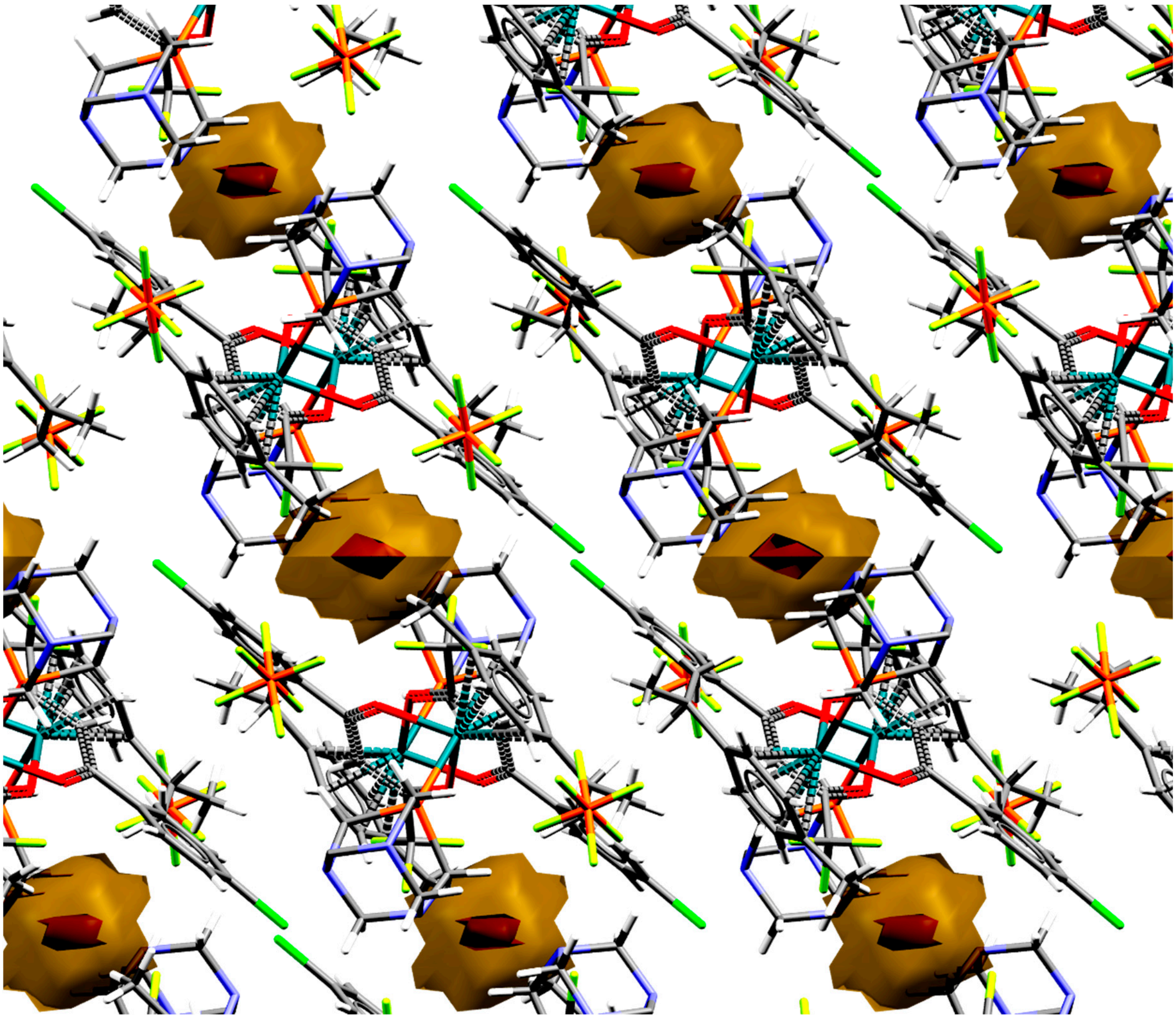

Figure 16. Packing of **6** along b axis. Solvent inaccessible voids are indicated with ochre clouds, solvent accessible voids are indicated with red clouds.
